# Management of Renal Cell Carcinoma—Current Practice in Sub-Saharan Africa

**DOI:** 10.15586/jkcvhl.2019.122

**Published:** 2019-12-02

**Authors:** Ayun Cassell, Mohamed Jalloh, Bashir Yunusa, Medina Ndoye, Mouhamadou M. Mbodji, Abdourahmane Diallo, Saint Charles Kouka, Issa Labou, Lamine Niang, Serigne M. Gueye

**Affiliations:** 1 Department of Urology and Andrology, Hopital General de Grand Yoff, Dakar, Senegal; 2 Department of Surgery, Liberia College of Physicians and Surgeons, Monrovia, Liberia; 3 UFR Sante, Universite de Thies, Thies, Senegal

**Keywords:** immuotherapy, management, radical nephrectomy, renal cell carcinoma, sub-saharan africa

## Abstract

There is a global variation in the incidence of renal masses with the developed nations having a greater incidence. About 80–90% of renal malignancies are renal cell carcinomas (RCC) which account for 2–4% of all cancers. In Africa and the Middle East, the age-standardized incidence for RCC is 1.8–4.8/100,000 for males and 1.2–2.2/100,000 for females. The management of renal cell cancer is challenging. A multidisciplinary approach is effective for diagnosis, staging, and treatment. Guidelines recommend active surveillance, thermal ablation, partial nephrectomy, radical nephrectomy, cytoreductive nephrectomy and immunotherapy as various modalities for various stages of RCC. However, open radical nephrectomy is most widely adopted as an option for treatment at various stages of the disease in sub-Saharan Africa due to its cost-effectiveness, applicability at various stages, and the reduced cost of follow-up. Nevertheless, most patients in the region present with the disease in the advanced stage and despite surgery the prognosis is poor.

## Introduction

There is a global variation in the incidence of renal masses with the developed nations having a greater incidence (1). GLOBOCAN has estimated that approximately 300,000 men and women were diagnosed with renal cancer globally and nearly 150,000 renal cancer-related deaths occurred in 2012 (2). In 2018, the global incidence was 403,262 with 175,098 deaths reported by GLOBOCAN (3). About 80–90% of renal malignancies are renal cell carcinomas (RCCs) which account for 2–4% of all cancers (4). In Africa and the Middle East, the age-standardized incidence for RCC is 1.8–4.8/100,000 for males and 1.2–2.2/100,000 for females (2). A review by Atanda and Haruna in 2017 estimated the incidence of RCC in Nigeria as approximately 0.3/100,000 of the population (5).

The morbidity and mortality of renal malignancy have reduced or remained steady in developed nations, but this finding cannot be postulated for the disease in sub-Saharan nations. Major risk factors and etiologies identified include smoking, hypertension, obesity, and familial RCC syndrome (1). However, moderate alcohol intake and consumption of fruits and vegetables have proven to have a protective action (6, 7).

The management of renal cell cancer is challenging. A multidisciplinary approach is effective for the diagnosis, staging, and treatment. Available guidelines from the American Urological Association (1), European Association of Urology (8), and Canadian Urological Association (9) show a consensus on partial nephrectomy, radical nephrectomy, anti-angiogenic/immunotherapy, and cytoreductive therapy as treatment options for RCC based on the staging. In 2015, Zekri and colleagues published a guideline for clear cell renal carcinoma for Middle East Countries and Africa emphasizing on a multidisciplinary board for renal malignancy due to the complexity of treatment options (4).

Whether the recommendations set by these guideline have been fully adopted into clinical practice in the sub-Saharan region remains unknown. It is relevant that a consensus on the management of renal masses is reached in a regional context that is clinically applicable. We therefore present findings from the Western sub-Saharan nations on the management of RCC to stimulate a development of a regional consensus.

## Methodology

A thorough search of the available literature was conducted by reviewing publications from 1996 to 2019 using the search engines PubMed, Google Scholar, African Journal Online, and Cochrane Library. The search terms used included “Management of Renal Masses” appended with the following indices: Guidelines, Africa, Sub-Saharan Africa, Senegal, Liberia, Ghana, Nigeria, Burkina, Mali, Togo, Ivory Coast, Kenya, Uganda. There were a total of 209 study results and only 30 studies were included in the review. These included clinical guidelines, systemic reviews and meta-analysis, randomized trials, prospective and retrospective studies. The review considered papers dealing with adult renal tumors only. All pediatric renal lesions were excluded.

Eleven published papers from the sub-Saharan region were included for cross-tabulation of data. Both the abstracts and full texts were reviewed for demographics, age, gender, duration of symptoms, clinical presentation, risk factors, associated complication, diagnostic imaging modalities, tumor consistency, tumor size, clinical staging, histological type, treatment option, rate of recurrence, and outcome. The results of the literature search are reflected in Tables 1–4. A quantitative analysis of the data retrieved is represented in the main text of the results using pool analysis of age, gender, duration of symptoms, and clinical presentation. The risk factors, complications, diagnostic modalities, and outcomes were analyzed qualitatively as depicted in the following section.

**Table 1:** Demographics, age distribution, and duration of symptoms in patients with renal cell carcinoma

Study	Number of patients	Gender distribution		Age distribution in years		Duration of symptoms in months	
		Male (%)	Female (%)	Mean age	Age range	Mean	Range
Tengue et al. (Togo) (10)	32	46.9	53.1	48.1 ± 10.8	31–75	8.6 ± 7.8	1–48
Fall et al. (Senegal) (11)	74	48.7	51.3	49	18–72	10	1–96
Gueye et al. (Senegal) (12)	42			51	18–83	14	
Ahmed et al. (Nigeria) (13)	61	37.4	62.6	44	15–70	12	1–192
Tijani et al. (Nigeria) (14)	64	37.5	62.5	41.8	20–75		
Mbaeri et al. (Nigeria) (15)	19	26.3	73.4	52.6 + 15.8	22–75		
Avakoudjo et al. (Benin) (16)	46	60.9	39.1	54	19–83		
Coulibaly et al. (Burkina Faso) (17)	24	41.7	58.3	48.2 ± 8.02	17–82		
Muhammed et al. (Nigeria) (18)	51	33.3	66.7	43.1			
Badmus et al. (Nigeria) (19)	18	61.6	38.4	47.5	16–80	43.6	2–104
Salako et al. (Nigeria) (20)	51	33.3	66.7	41.7	21–83		6–32

**Table 2:** Clinical manifestation, risk factors, complications, and diagnostics modalities of renal cell carcinoma

Study	Clinical presentation			Risk factors	Associated complications	Diagnostic modalities	Imaging findings of tumors
	Flank pain (%)	Hematuria (%)	Lumbar mass (%)				
Tengue et al.	90.6	28.1	9.3	Smoking, hypertension	Severe anemia	CT-scan, ultrasound	Solid: 93.7%; cystic: 6.3%
Fall et al.	87.8		77				
Gueye et al.			70.8			Ultrasound, IVU	
Ahmed et al.	74.9	57.9	86				
Tijani et al.	86	40.6	90	Family history, smoking, dyes exposure	Anemia, pedal edema, Varicocele	CT-Scan	Solid: 100%
Mbaeri et al.	78.9	522.6	78.9			Ultrasound, IVU	
Avakoudjo et al.	100		48.4	Hypertension	Anemia, Varicocele	CT-scan, ultrasound, IVU	
Coulibaly et al.			66.7				
Badmus et al.	94.4	50.0	83.3		Anemia, pleural effusion	Ultrasound, IVU	
Salako et al.	7.9	11.8	37.2%	Hypertension, smoking, obesity		CT-scan, ultrasound, IVU	

CT-scan: computed tomography scan; IVU: intravenous urography.

**Table 3:** Renal tumor size, clinical staging, and commonest histological types in sub-Saharan Africa

Study	Incidental finding (%)	Tumor size in centimeters	Range	Clinical staging	Locally advanced	Advanced/metastatic	Commonest histological types
		Mean		Localized (%)			
Tengue et al.	3.1	12.8±4.3	5–25	68.8	9.4	21.8	93.1% (clear cell RCC)
Fall et al.	2.7	12	2.4–26	T2 (39.2)	T3 (33.7)	31	63.5% (RCC)
Gueye et al.						25	93% (RCC)
Ahmed et al.				30.1	63.9	60.3	59% (clear cell RCC)
Tijani et al.	1.5	22	12–30	6.3	60.9	36	60% (clear cell RCC)
Mbaeri et al.				21.1	21.1	57.8	53.8% (clear cell RCC)
Avakoudjo et al.				T2 (43.3)	T3 (13.3)	T4 (43.3)	58.8% (clear cell RCC)
Coulibaly et al.		13.6±5.8	7.8–21.1	40		40	95.8% (RCC)
Muhammed et al.	11.8			11.8		90	74.5% (clear cell RCC)
Badmus et al.				11.1		88.9	72.2% (RCC) 33% (clear cell RCC)
Salako et al.	5.9			47.1	35.3	17.6	60.8% (clear cell RCC)

RCC: renal cell carcinoma.

**Table 4:** Treatment modalities for renal tumors and outcome of the disease and treatment in sub-Saharan Africa

Study	Treatment options			Others	Rate of recurrence	Outcome
	Radical nephrectomy (%)	Partial nephrectomy (%)	Anti-angiogenics			
Tengue et al.	90.6		9.4% (Sunitinib)	6.9% (LND)	9.4%	28.1% (mortality)
Fall et al.	58.1	1.4			12.2%	47.3% mortality
Gueye et al.	60			40% received no Rx due to advanced disease		38% (1-year mortality)
Ahmed et al.						20 months (median survival)
Tijani et al.	70.3		29.6% (bevacizumab, interferon alpha)	4 unresectable tumors		All T4 and M1 were dead within a year
Mbaeri et al.	57.9	5.3		1 unresectable tumor		Average follow-up (4 months)
Avakoudjo et al.	56.5					8.7% perioperative mortality, most lost to follow-up
Coulibaly et al.	100					
Muhammed et al.	100		7.8% adjuvant immunotherapy			90% died within 1-year of nephrectomy
Badmus et al.	72.2			11% unresectable tumor		7.6% mortality
Salako et al.	78.4		7.8% sunitinib/sorafenib			

LND: lymph node dissection.

## Results from the Retrieved Literature

There were 11 published papers from the sub-Saharan region (10–20) including Senegal, Togo, Nigeria, and Benin which discussed the management of renal tumors. A total of 482 cases of renal masses were obtained from the 11 studies (Table 1). Based on a pool analysis of the data, there was a predominance of renal masses amongst females (57.2%) as opposed to males (42.8%) in sub-Saharan Africa. Benin (16) and Nigeria (19) contrarily reported a greater male to female ratio. The mean age of diagnosis was 47.4 years with an age range of 15–83 years. Pooled results from the reviewed data showed a mean duration of symptoms from onset to presentation at 17.6 months with a range of 1–192 months. Most published literature in West Africa reported flank pain, hematuria, and lumbar mass as the commonest clinical presentation with lumbar mass being highlighted in all the studies reviewed (10–17, 19, 20). Smoking (10, 20), hypertension (10, 16, 20), obesity (20), family history (14), and exposure to industrial dyes (14) were the risk factors associated with renal malignancy reported from the sub-Saharan literature (Table 2). The data have shown that most patients in sub-Saharan Africa presented with locally advanced to metastatic disease. Only a few studies from Togo (10) and Nigeria (20) showed a higher percentage of patients presenting with clinically localized disease (68.8% and 47.1%, respectively). Anemia, varicocele, hypertension, and pleural effusion were common renal cancer-related complications reported by Tengue et al. (10), Tijani et al. (14), and Salako et al. (20). Histological reports from sub-Saharan nations revealed that RCC was the commonest histological type of cancer with clear cell carcinoma being the most dominant variant at an average of 61.6% (Table 3). The average tumor size from four publications (10, 11, 14, 17) was 15.1 cm with a range of 2.4–30 cm. An average of 74.4% of patients presenting with renal masses were managed with radical nephrectomy. However, data from Senegal and Nigeria revealed 1.4% and 5.3% of renal masses were managed with partial nephrectomy. Reports from Togo (10) and Nigeria (14, 18, 20) mentioned the use of immunotherapy in some patients with advanced disease. Sunitinib, bevacizumab, interferon alpha, and sorafenib were the immunotherapeutic options available in these studies (Table 4). A study from Senegal by Gueye et al. (12) reported that 40% of patients could not receive treatment due to the disease being in the advanced stage while Tijani et al. (14), Mbaeri et al. (15), and Badmus et al. (19), reported patients with unresectable tumor during laparotomy.

## Discussion of the Recommended Standard of Care

### Diagnosis

**Figure 1:**
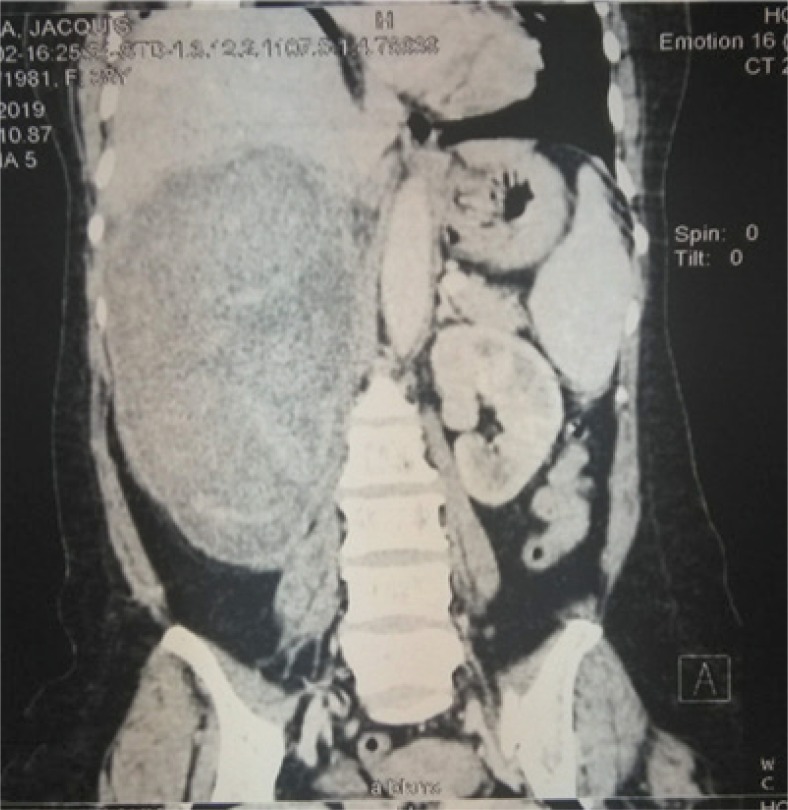
A contrast coronal CT-scan showing a hyper vascularized right T4 renal tumor found to have affected the renal vein, IVC, and inferior margin of the liver.

**Figure 2:**
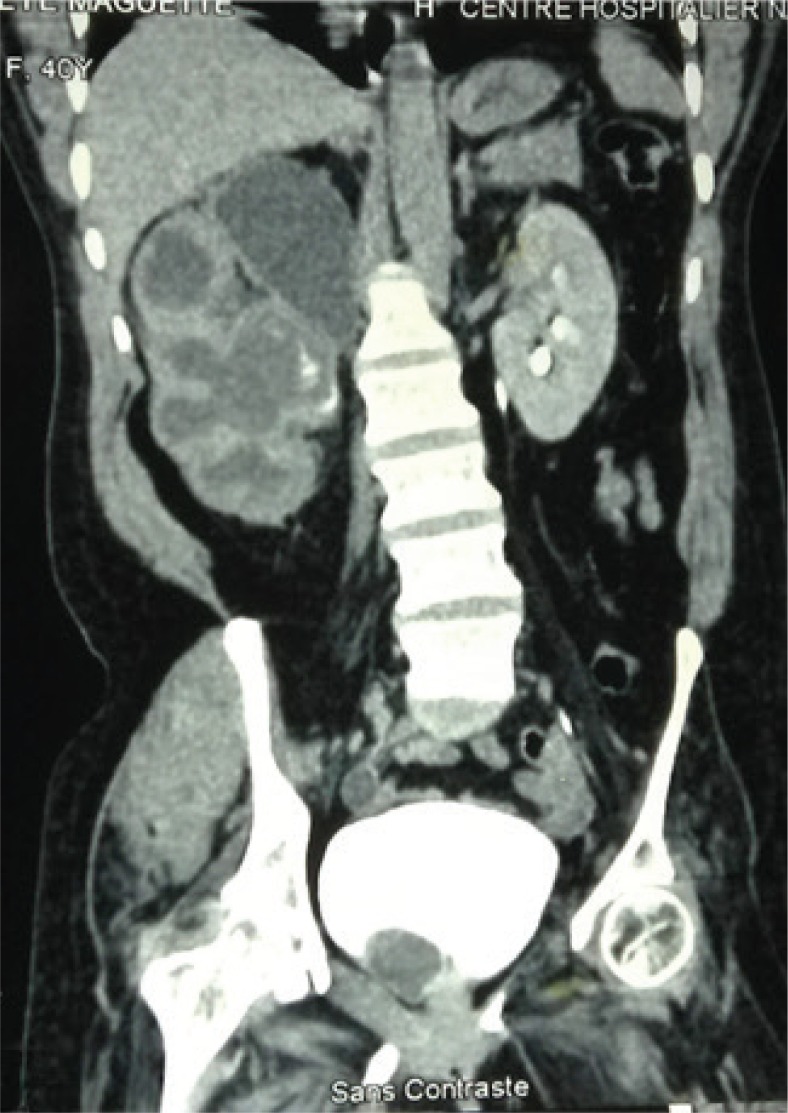
A contrast CT-scan showing a cystic right renal tumor in the upper pole with associated hydronephrosis. Courtesy: Grand Yoff Hospital.

History and physical exam are important components of assessing a renal mass. The relevant clinical presentation including hematuria, flank pain, and lumbar mass should be ascertained. All possible risk factors or etiologies should be sought. Physical exam findings of varicocele or pedal edema could depict vascular involvement of the tumor or inferior vena cava (IVC) invasion. Though most authors reported the clinical triad of RCC, the presence of a lumbar mass was reported in all the studies reviewed from the sub-Saharan region (10–20). A complete blood count (CBC), renal function (creatinine, estimated glomerular filtration rate), liver function (alanine transaminase, aspartate aminotransferase), and bony markers (alkaline phosphatase, calcium) should be evaluated (1, 9). In the presence of an elevated creatinine, a renal scintigraphy should be performed to assess renal function. Computed tomographic scan (CT-scan) is the imaging of choice with about 90% accuracy for renal masses (Figures 1 and 2). Renal malignancy is most likely when contrast attenuation of 10–20 Hounsfield Unit (HU) is obtained for a renal mass (9). CT scan is essential for staging renal cancer, lymph node assessment, as well as identification of metastasis. A chest CT is ideal for assessing metastasis when findings of chest x-ray are equivocal. Magnetic resonance imaging (MRI) and Doppler ultrasound are useful for determining the IVC involvement. Findings by Tengue et al., Tijani et al., Avokoudjo et al. and Salako et al. highlighted the use of CT-scan and ultrasound to diagnose and stage renal malignancies. The use of intravenous urogram (IVU) though limited was reported by some studies (12, 15, 19, 20) from the sub-Saharan region. It may have been useful for large tumors that may have distorted the renal parenchyma.

CT-scan or MRI findings of cystic renal lesion, Bosniak class I and class II, have minimum risk of malignancy and require no follow-up. However, Bosniak class IIF has a 10% risk of malignancy and follow-up with ultrasound or CT is recommended. Bosniak class III and IV have 65% and 92% risk of malignancy respectively and require treatment (Figure 2) (9).

The use of renal mass biopsy is optional and should be performed if the histological results will change the decision of the management. Renal mass biopsy is essential for patients with metastatic disease or hematological malignancy (lymphoma) (1) who are candidates for systemic therapy. The percutaneous ultrasound or CT-guided core biopsy approach has proven to be safe (1, 9). The sensitivity and specificity of renal mass biopsy for the diagnosis of renal malignancy are high, especially for RCC (21).

## Management of Localized Renal Cell Carcinoma (cT1-T2, N0 M0)

### cT1a

It is recommended that patients with cT1a renal tumors should be offered partial nephrectomy as it is associated with good oncological outcomes and minimizes the risk of chronic kidney disease (1). Partial nephrectomy or nephron sparing procedures should be considered for patients with bilateral tumors, tumors of solitary kidney, or patients with familial renal tumor. However, in candidates undergoing partial nephrectomy, a negative surgical margin should remain a priority. More complex tumors not amenable to partial nephrectomy can undergo radical nephrectomy. In elderly patients who are frail and cannot withstand surgery, thermal ablation of the renal tumor is an option but a biopsy is required before the procedure. Active surveillance has been advocated for select patients but regular follow-up with imaging for 3–6 months is warranted.

### cT1b

Tumor greater than 4 centimeters (cm) but <7 cm is amenable to partial nephrectomy either open, laparoscopic, or robotic as evidenced by the acceptable oncological results (9). When partial nephrectomy is not technically feasible, a laparoscopic radical nephrectomy should be considered over open radical nephrectomy as it is associated with less postoperative pain and better recovery (22, 23). Active surveillance has shown some benefits in selected patients with RCC following renal mass biopsy. Ablative therapy should not be considered an option in this subset as complete thermal ablation is challenging in tumors > 4 cm (9).

### cT2

Patients with renal tumor >7 cm limited to the kidney can undergo open or laparoscopic radical nephrectomy (24, 25). Extended partial nephrectomy is discouraged in this population (9).

It may be debatable that patients from the sub-Saharan settings are offered partial nephrectomy for localized disease as the technique itself has not been widely applied amongst urologists in the region. The lack of resources, cost of active surveillance, and the loss to follow-up of most patients in these settings render the nephron-sparing procedure less desirable. Radical nephrectomy may provide a better oncological result though the risk of chronic kidney disease is not eliminated.

## Management of Locally Advanced Renal Cell Carcinoma (T1-T2, N1, M0 or T3, N0/N1, M0)

### T3

Approximately 4–10% of RCCs will involve the IVC (9, 26). Tumors >7 cm that have not invaded the Gerota fascia but involve the IVC are managed with radical nephrectomy and thrombectomy as this approach has provided acceptable morbidity in the absence of metastasis (9, 26). When there is an upper pole tumor, ipsilateral involvement of the adrenal gland intraoperatively or following imaging, an ipsilateral adrenalectomy is ideal as 1.9–7.5% of renal malignancy will involve the ipsilateral adrenal gland (27). Routine regional lymphadenectomy is not recommended in localized disease but patients with clinical (N1, M0) disease are candidates for regional lymph node dissection. The role of neoadjuvant and adjuvant therapy for RCC remains unclear.

The evidence has shown that most patients in sub-Saharan Africa present with locally advanced to metastatic disease (11–19). Open radical nephrectomy has been well adopted for locally advanced disease in Africa. Evidence from published literature in the region has shown that an average of 74.4% of patients presenting with renal masses were managed with radical nephrectomy. A 16-year retrospective study by Tengue et al. (10) in Togo involving 32 patients with RCC who underwent surgery showed that 6.9% of patients required lymph node dissection.

## Treatment of Advanced Disease (T4 N0/N1 M0)

### T4

Surgical resection is the only available treatment option for T4 tumors. These tumors are best managed in high-volume centers with resection of the ipsilateral adrenal gland and part of the liver, pancreas, or diaphragm if required (9). Most of these patients already have occult lymph node involvement which demands regional lymph node dissection. Despite these gestures, the 5-year overall survival is poor, and the surgical morbidity of extensive resection should be weighed against the oncological benefits (28, 29).

Studies by Tijani et al. (14) and Muhammed et al. (18) from Nigeria showed a poor prognosis of T4 disease despite intervention with an overall 1-year disease survival of less than 10%.

## Treatment of Metastatic Disease (Any T, Any N + M1)

Cytoreductive nephrectomy is recommended in patients with metastatic disease. Studies have provided better results when nephrectomy is combined with systemic therapy as compared to systemic therapy alone (30). Cytoreductive nephrectomy and interferon alpha help to improve survival rates in patients with RCC (8). In well-selected patients, metastasectomy following nephrectomy has given good results. Metastasis to the pancreas, lungs, bone, and adrenal gland has a more favorable prognosis (9). Radiotherapy to a local or distant site of metastasis (brain, bone) in patients with RCC can relieve pain. Chemotherapy plays a limited role as a systemic therapy in RCC, especially clear cell metastatic RCC. However, Gemcitabine, 5- Fluorouracil, and Doxorubicin have some documented effect (8).

### Immunotherapy

Data have shown that interferon alpha and Bevacizumab increase the response rate and progression free survival in metastatic RCC compared to interferon alpha alone (8). Ipilimumab plus nivolumab in treatment-naïve patients with clear cell RCC provide a better survival rate compared to sunitinib (8). These drugs are associated with significant side effects and should be administered by a multidisciplinary team.

The use of systemic therapy following cytoreductive nephrectomy in patients with advanced or metastatic RCC is not well-documented in West Africa. However, a few studies from Togo (10) and Nigeria (14, 18, 20) documented the use of immunotherapy in some patients with advanced disease. Sunitinib, bevacizumab, interferon alpha, and sorafenib were the available immunotherapeutic options for these studies. A study by Muhammed et al. documented that 4 (7.8%) patients who had adjuvant treatment (immunotherapy or vascular endothelial growth factor (VEGF)-tyrosine kinase inhibitors) had a better prognosis (18). The management of advanced and metastatic disease was not well-documented in the review from sub-Saharan nations. Moreover, most of the institutions lack the recommended immunotherapeutic agents or rather referred the patients to medical oncology for further management. It is important that urologists and oncologists from sub-Saharan Africa coordinate with relevant pharmaceutical companies for provision or supply of these agents at an affordable cost.

### Follow-up

Post-surgical follow-up should be based on individual risk assessment. Low-risk patients should have imaging (CT, MRI, or ultrasound) performed within a year following surgery. Chest X-ray should be performed yearly for the first 3–6 years to check for metastasis. Moderate- to high-risk patients will require an MRI or CT-scan 6 months after surgery. A yearly chest X-ray or chest CT-scan is advisable for up to 5 years.

### Outcome

Data from the review showed heterogenous results on the perioperative mortality following radical nephrectomy in the sub-Saharan region. However, analysis of reports from Nigeria (14, 19), Togo (16), and Mali (17) showed a perioperative mortality of 5.1% following radical nephrectomy. Most of these deaths were due to perioperative hemorrhage or pulmonary complications. These data are comparable to the results of a systemic review of RCC in Nigeria which showed a perioperative mortality of radical nephrectomy ranging from 6.3% to 7.8% (5). These figures are much higher than the perioperative mortality of radical nephrectomies in Europe and North America which is about 2.8% (5). The late patient presentation, the lack of expertise for nephron sparing procedures, and the underequipped intensive care unit have contributed to this dismal result. However, more funding, quality imaging, and diagnostics as well as human expertise are required for uro-oncological care in the region with promotion of a multidisciplinary approach. Urologists, medical oncologists, radio-oncologists, and radiologists should communicate to provide the best standard of care and adopt a guideline that is practical, feasible but evidence based. This is only achievable through proper documentation and coordinated research in the African context.

Extrapolation of the 5-year overall survival of RCC following treatment is challenging as the reportings are heterogenous. Nevertheless, a retrospective review of 61 patients with RCC in Nigeria had a 5-year survival of 46% for young adults and 26% for older adults using the Kaplan–Meier analysis (13). Another contemporary series in Nigeria showed a lower 5-year survival of RCC of less than 10% following radical nephrectomy (18). Better cancer-awareness programs, maintaining an effective cancer-treatment guideline, and establishing a cancer registry in the sub-region are required to achieve 5-year overall survival rates of 55% and 73% as obtained from Europe and the United States, respectively (5).

## Conclusion

The management of renal cell cancer is challenging. A multidisciplinary approach is effective for diagnosis, staging, and treatment. Active surveillance, thermal ablation, partial nephrectomy, radical nephrectomy, cytoreductive nephrectomy, and immunotherapy are being recommended as various modalities by guidelines for various stages of RCC. However, open radical nephrectomy is most widely adopted as a treatment option at various stages of the disease in sub-Saharan Africa due to its cost-effectiveness, applicability at various stages, and the reduced cost of follow-up. Nevertheless, most patients present with advanced disease in the region and despite surgery the prognosis is poor.

## Conflict of Interest

The authors declare no conflict of interest regarding this article.
